# Autophagy is essential for hearing in mice

**DOI:** 10.1038/cddis.2017.194

**Published:** 2017-05-11

**Authors:** Chisato Fujimoto, Shinichi Iwasaki, Shinji Urata, Hideaki Morishita, Yuriko Sakamaki, Masato Fujioka, Kenji Kondo, Noboru Mizushima, Tatsuya Yamasoba

**Affiliations:** 1 Department of Otolaryngology, Faculty of Medicine, The University of Tokyo, 7-3-1, Hongo, Bunkyo-ku, Tokyo 113-8655, Japan; 2 Department of Biochemistry and Molecular Biology, Graduate School and Faculty of Medicine, The University of Tokyo, 7-3-1, Hongo, Bunkyo-ku, Tokyo 113-0033, Japan; 3 Research Center for Medical and Dental Sciences, Tokyo Medical and Dental University, 1-5-45, Yushima, Bunkyo-ku, Tokyo 113-8519, Japan; 4 Department of Otolaryngology Head and Neck Surgery, Keio University, 35, Shinanomachi, Shinjuku-ku, Tokyo 160-8582, Japan

## Abstract

Hearing loss is the most frequent sensory disorder in humans. Auditory hair cells (HCs) are postmitotic at late-embryonic differentiation and postnatal stages, and their damage is the major cause of hearing loss. There is no measurable HC regeneration in the mammalian cochlea, and the maintenance of cell function is crucial for preservation of hearing. Here we generated mice deficient in autophagy-related 5 (*Atg5*), a gene essential for autophagy, in the HCs to investigate the effect of basal autophagy on hearing acuity. Deletion of *Atg5* resulted in HC degeneration and profound congenital hearing loss. In autophagy-deficient HCs, polyubiquitinated proteins and p62/SQSTM1, an autophagy substrate, accumulated as inclusion bodies during the first postnatal week, and these aggregates increased in number. These findings revealed that basal autophagy has an important role in maintenance of HC morphology and hearing acuity.

Hearing loss is the most common sensory disorder in humans, affecting all generations^1^ with 360 million people with disabling hearing loss worldwide.^2^ The main cause of hearing loss is damage to auditory hair cells (HCs), which is caused by different etiologies such as aging, exposure to intense noise, ototoxic medications, and genetic disorders. Auditory HCs are postmitotic at late-embryonic differentiation and postnatal stages and are located in the organ of Corti, a core component of the cochlea (the auditory end organ of the inner ear). Because mammalian auditory HCs are not spontaneously replaced after their loss in the adult cochlea,^3, 4^ hearing loss caused by HC loss is irreversible. Investigations using multiple strategies to regenerate auditory HCs, such as drug, cell, and gene therapies, have thus far shown that their effects are very limited and insufficient to restore hearing function.^5, 6, 7^ Therefore, maintenance of existing HC function is crucial for preservation of hearing.

Autophagy is an intracellular system where cytoplasmic components are delivered to and degraded in the lysosome. Macroautophagy is one type of autophagy that is initiated by the formation of a sequestering structure, called the phagophore or isolation membrane.^8^ The phagophore matures into a closed double-membrane-bound structure, termed the autophagosome, which then fuses with a lysosome. The inner membrane of the autophagosome and the materials contained within are then degraded by lysosomal hydrolases. Autophagy is typically induced by starvation as well as other stresses, and basal autophagy is also important for quality control of cytoplasmic components and homeostasis of various postmitotic cells, such as neurons, hepatocytes, and cardiomyocytes.^9^

As the auditory HCs are postmitotic and long lived, we hypothesized that basal autophagy in these cells may have an important role in the acquisition and/or preservation of hearing function in mammals. Here we report that loss of autophagy in the auditory HCs leads to damage to these cells. Mice deficient in autophagy-related 5 (*Atg5*), a gene essential for autophagy,^10^ in the auditory HCs develop profound congenital hearing loss and degeneration of the HCs. We revealed that intracellular quality control by basal autophagy in the HCs has an important role in hearing acuity and maintenance of cell morphology.

## Results

### Basal autophagy flux detected in auditory HCs

**Figure 1:**
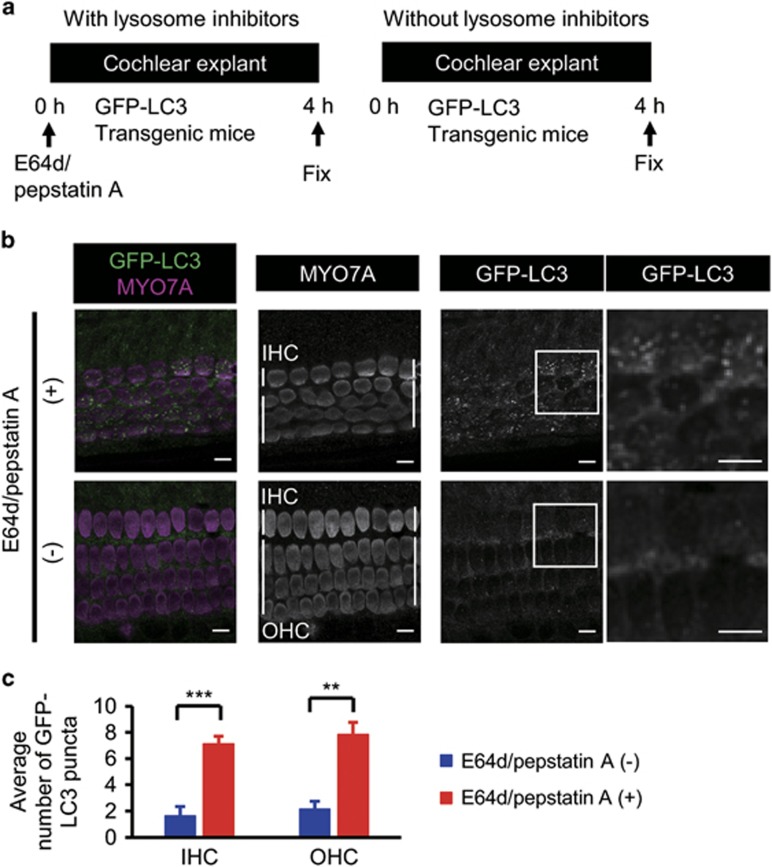
Basal autophagy flux was detected in auditory HCs. (**a**) Experimental design after the administration of lysosomal protease inhibitors, E64d and pepstatin A, to cochlear explant cultures established from P5 GFP-LC3 transgenic mice. Fixation was performed 4 h after the administration of lysosomal protease inhibitors. (**b**) Basal autophagy flux in the auditory HCs. GFP-LC3 puncta in the HC cytoplasm in a cochlear explant culture established from P5 GFP-LC3 transgenic mice were monitored. The accumulation of GFP-LC3 puncta was increased in cultures treated with E64d and pepstatin A for 4 h in comparison with cultures without these inhibitors. MYO7A was used as a marker of the HC cytoplasm. Magnified images of GFP-LC3 staining are shown in the rightmost panels. IHC, inner HC; OHC, outer HC. Scale bars: 10 *μ*m. (**c**) Quantitative analysis of the accumulation of GFP-LC3 puncta. The average number of GFP-LC3 puncta was significantly increased in cultures treated with E64d and pepstatin A (*n*=5) in comparison with cultures without these inhibitors (*n*=5). Error bars: S.E. **P<0.01, ***P<0.001

First, we aimed to examine basal autophagy flux in the auditory HCs. We monitored green fluorescence protein (GFP)-labeled microtubule-associated protein 1 light chain 3b (LC3) puncta, which represent autophagy-related structures, in the HC cytoplasm in a cochlear explant culture established from postnatal day 5 (P5) GFP-LC3 transgenic mice (Figure 1a).^11^ GFP-LC3 mice express a fusion cDNA encoding enhanced GFP jointed at its C-terminus to rat LC3 under the control of the CAG promoter. The occurrence of autophagy in mouse tissues can be monitored by the GFP. In both the inner and outer HCs, only a small number of GFP-LC3 puncta were observed. However, the number of GFP-LC3 puncta increased after a 4-h treatment with the lysosomal protease inhibitors E64d and pepstatin A (Figures 1b and c). These data suggest that autophagosomes are continuously turned over dependently on lysosomal function in both the inner and outer HCs (Figure 1).

### Generation of Atg5^flox/flox^;Pou4f3-Cre mice

**Figure 2:**
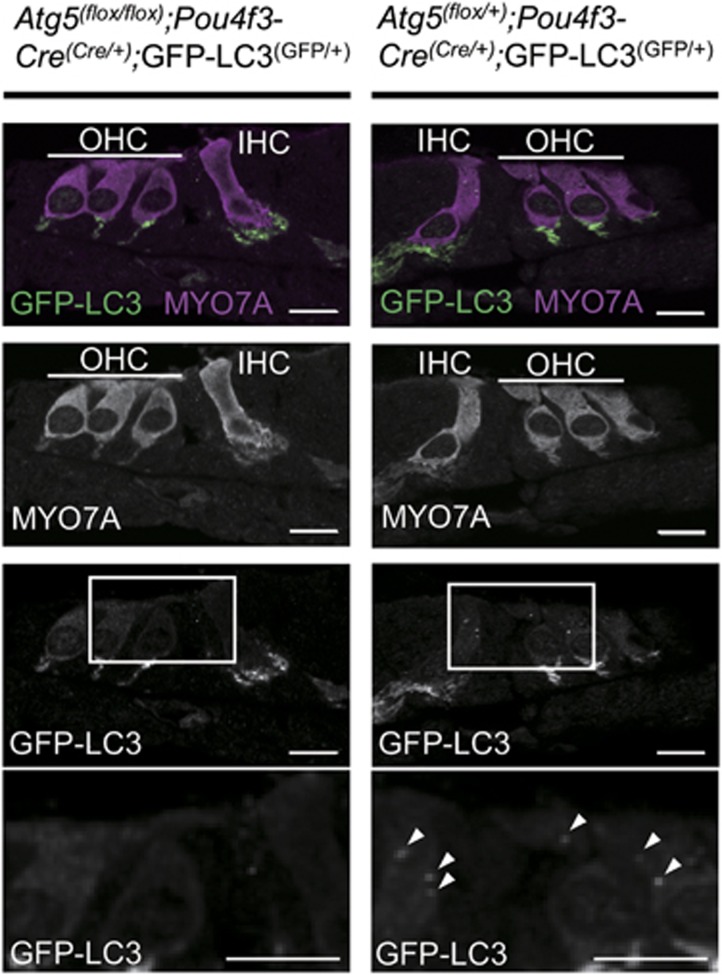
Autophagy was inhibited in the HCs from *Atg5*^*flox/flox*^*;Pou4f3-Cre* mice. In the HCs from P5 *Atg5*^*flox/+*^*;Pou4f3-Cre*;GFP-LC3 mice, a number of GFP-LC3 puncta (white arrow heads) were observed in the cochlear HCs. Conversely, P5 *Atg5*^*flox/flox*^*;Pou4f3-Cre*;GFP-LC3 mice showed almost no GFP-LC3 puncta in the HCs. MYO7A was used as a marker of the HC cytoplasm. Magnified images of GFP-LC3 staining are shown in the lowermost panels. The GFP-positive region was also observed at the auditory nerve fibers in both *Atg5*^*flox/flox*^*;Pou4f3-Cre*;GFP-LC3 and *Atg5*^*flox/+*^*;Pou4f3-Cre*;GFP-LC3 mice. The possibility that this region indicated autophagy-related structures or aggregates could not be denied. IHC, inner HC; OHC, outer HC. Scale bars: 10 *μ*m

Next, we generated mice deficient in *Atg5* in auditory HCs to investigate the physiological function of basal autophagy in these cells. Mice bearing an *Atg5*^*flox*^ allele^12^ were crossed with POU domain, class 4, transcription factor 3 (*Pou4f3*)-*Cre* transgenic mice.^13^ To confirm the inhibition of autophagy in the HCs, we crossed these mice with GFP-LC3 transgenic mice.^11^ In the control auditory HCs from P5 *Atg5*^*flox/+*^*;Pou4f3-Cre*;GFP-LC3 mice, a number of GFP-LC3 puncta were observed (Figure 2), suggesting that autophagy is constitutively active. Conversely, P5 *Atg5*^*flox/flox*^*;Pou4f3-Cre*;GFP-LC3 mice showed almost no GFP-LC3 puncta in the HCs. These results suggest that autophagosome formation is suppressed in the *Atg5*-deficient HCs.

### Aggregates containing ubiquitin and p62 observed in Atg5-deficient HCs

**Figure 3:**
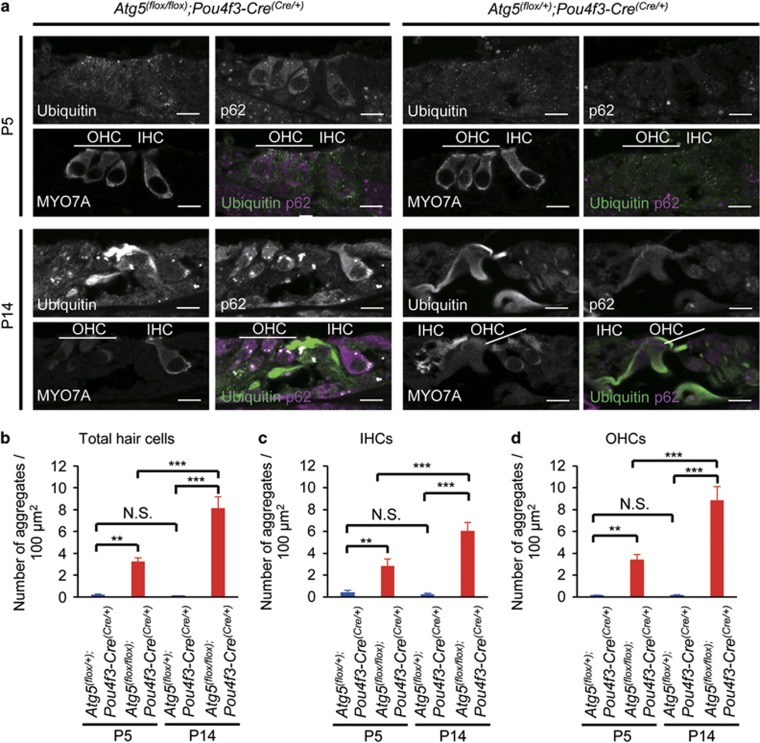
Aggregates containing ubiquitin and p62 were observed in the HCs from *Atg5*^*flox/flox*^*;Pou4f3-Cre* mice. (**a**) Aggregates in the HCs from *Atg5*^*flox/flox*^*;Pou4f3-Cre* mice. In the HCs from P5 *Atg5*^*flox/+*^*;Pou4f3-Cre* mice, punctate signals positive for ubiquitin and/or p62 were not observed. In the HCs from P5 *Atg5*^*flox/flox*^*;Pou4f3-Cre* mice, aggregates containing ubiquitin and p62 were observed. At P14, these aggregates in *Atg5*^*flox/flox*^*;Pou4f3-Cre* mice became more massive. These additional aggregates were not generated in the HCs from *Atg5*^*flox/+*^*;Pou4f3-Cre* mice. MYO7A was used as a marker of the HC cytoplasm. IHC, inner HC; OHC, outer HC. Scale bars: 10 *μ*m. (**b–d**) Quantitative analyses of the number of aggregates containing ubiquitin and p62 for the total HCs (**b**), the IHCs (**c**), and the OHCs (**d**). The number of aggregates in P5 *Atg5*^*flox/flox*^*;Pou4f3-Cre* mice (*n*=6) was significantly greater than that in P5 *Atg5*^*flox/+*^*;Pou4f3-Cre* mice (*n*=6) for the total HCs (**b**), the IHCs (**c**), and the OHCs (**d**). The number of aggregates in P14 *Atg5*^*flox/flox*^*;Pou4f3-Cre* mice (*n*=6) was significantly greater than those in P14 *Atg5*^*flox/+*^*;Pou4f3-Cre* mice (*n*=6) and P5 *Atg5*^*flox/flox*^*;Pou4f3-Cre* mice for the total HCs (**b**), the IHCs (**c**), and the OHCs (**d**). Error bars: S.E. NS, not significant, **P<0.01, ***P<0.001

Because autophagy has an important role in intracellular quality control, dysfunction of autophagy gives rise to the accumulation of abnormal ubiquitinated proteins and organelles.^8^ Therefore, we examined whether polyubiquitinated proteins and p62/SQSTM1, which is an autophagy-specific substrate and accumulates under autophagy-deficient conditions,^14, 15^ were accumulated in the *Atg5*-deficient HCs. In the control HCs from P5 *Atg5*^*flox/+*^*;Pou4f3-Cre* mice, punctate signals positive for ubiquitin and/or p62 were rarely observed (Figures 3a and b). However, in the HCs from P5 *Atg5*^*flox/flox*^*;Pou4f3-Cre* mice, aggregates containing ubiquitin and p62 were observed (Figures 3a and b), suggesting that the failure of intracellular quality control by the dysfunction of basal autophagy started before the maturation of the organ of Corti. At P14, these aggregates increased in number in the *Atg5*-deficient HCs (Figures 3a and b). These aggregates were rarely generated in the HCs from *Atg5*^*flox/+*^*;Pou4f3-Cre* mice at P14 (Figures 3a and b). Quantitative analysis showed that the number of aggregates containing ubiquitin and p62 in the HCs of P14 *Atg5*^*flox/flox*^*;Pou4f3-Cre* mice was significantly greater than those in P14 *Atg5*^*flox/+*^*;Pou4f3-Cre* and P5 *Atg5*^*flox/flox*^*;Pou4f3-Cre* mice (Figure 3b). The progressive accumulation of aggregates in the *Atg5*^*flox/flox*^*;Pou4f3-Cre* mice was confirmed both in the inner and outer HCs (Figures 3c and d) and observed in every cochlear turn (Supplementary Figure S1). These results suggest that basal autophagy in the auditory HCs is important for preventing the accumulation of abnormal aggregates before the onset of hearing.

### Atg5^flox/flox^;Pou4f3-Cre mice developed congenital severe hearing loss and HC damage

**Figure 4:**
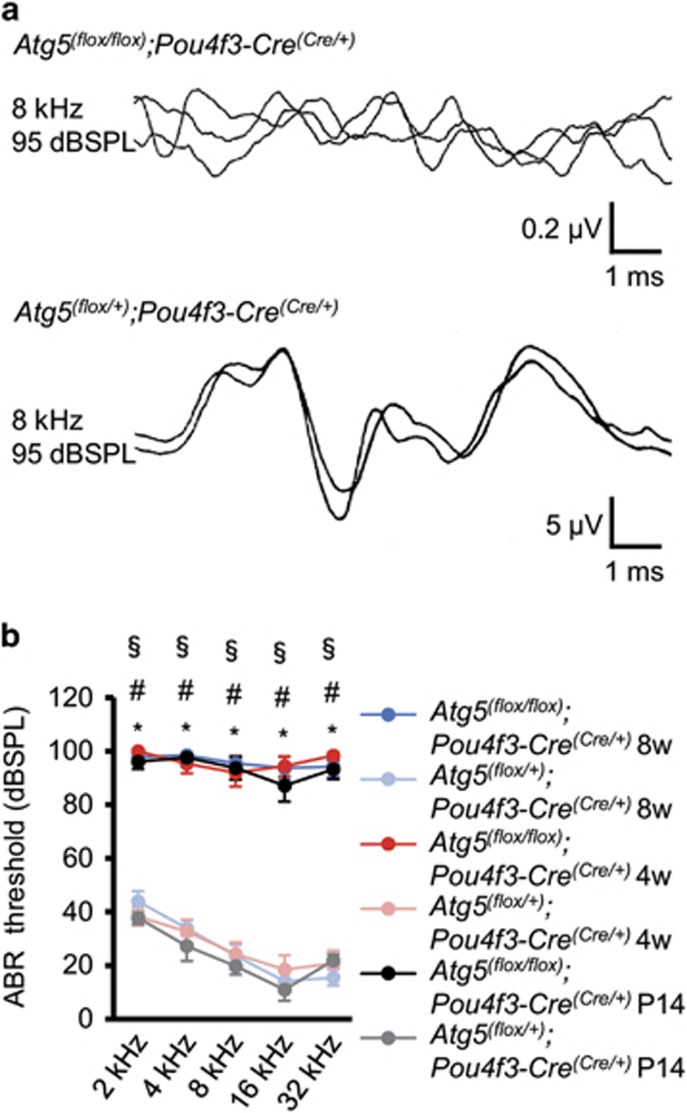
*Atg5*^*flox/flox*^*;Pou4f3-Cre* mice developed congenital severe hearing loss. (**a**) ABR waveform recordings over 10 ms to 8-kHz tone-burst stimuli from representative *Atg5*^*flox/flox*^*;Pou4f3-Cre* mice and *Atg5*^*flox/+*^*;Pou4f3-Cre* mice at P14. *Atg5*^*flox/flox*^*;Pou4f3-Cre* mice showed reproducible waveforms and *Atg5*^*flox/flox*^*;Pou4f3-Cre* mice showed no waveforms at 95 dB sound pressure level (dBSPL). (**b**) A comparison of the ABR threshold between *Atg5*^*flox/flox*^*;Pou4f3-Cre* and *Atg5*^*flox/+*^*;Pou4f3-Cre* mice was performed by Student’s *t*-test at P14, 4 weeks of age, and 8 weeks of age. When no response was obtained, we used 100 dBSPL as the threshold volume for calculations. *Atg5*^*flox/flox*^*;Pou4f3-Cre* mice showed profound congenital hearing loss already at P14 and thereafter and had significantly elevated hearing thresholds compared with *Atg5*^*flox/+*^*;Pou4f3-Cre* mice. The following genotypes were tested at P14: *Atg5*^*flox/flox*^*;Pou4f3-Cre* (*n*=6) and *Atg5*^*flox/+*^*;Pou4f3-Cre* (*n*=10); at 1 month: *Atg5*^*flox/flox*^*;Pou4f3-Cre* (*n*=6) and *Atg5*^*flox/+*^*;Pou4f3-Cre* (*n*=6); and at 2 months: *Atg5*^*flox/flox*^*;Pou4f3-Cre* (*n*=6) and *Atg5*^*flox/+*^*;Pou4f3-Cre* (*n*=8). Error bars: S.E. *P<0.001 for the study at P14; ^#^*P*<0.001 for the study at 4 weeks of age; ^§^*P*<0.001 for the study at 8 weeks of age

To assess the effect of basal autophagy on hearing acuity, we recorded auditory brainstem responses (ABRs) in *Atg5*^*flox/flox*^*;Pou4f3-Cre* mice. The ABR threshold of *Atg5*^*flox/flox*^*;Pou4f3-Cre* mice was extremely high and significantly elevated compared with that of *Atg5*^*flox/+*^*;Pou4f3-Cre* mice at every tested frequency at P14, 4 weeks, and 8 weeks of age (Figures 4a and b). These results suggest that *Atg5* deficiency leads to congenital profound hearing loss and that *Atg5* in the cochlear HCs is required for gaining normal hearing acuity.

**Figure 5:**
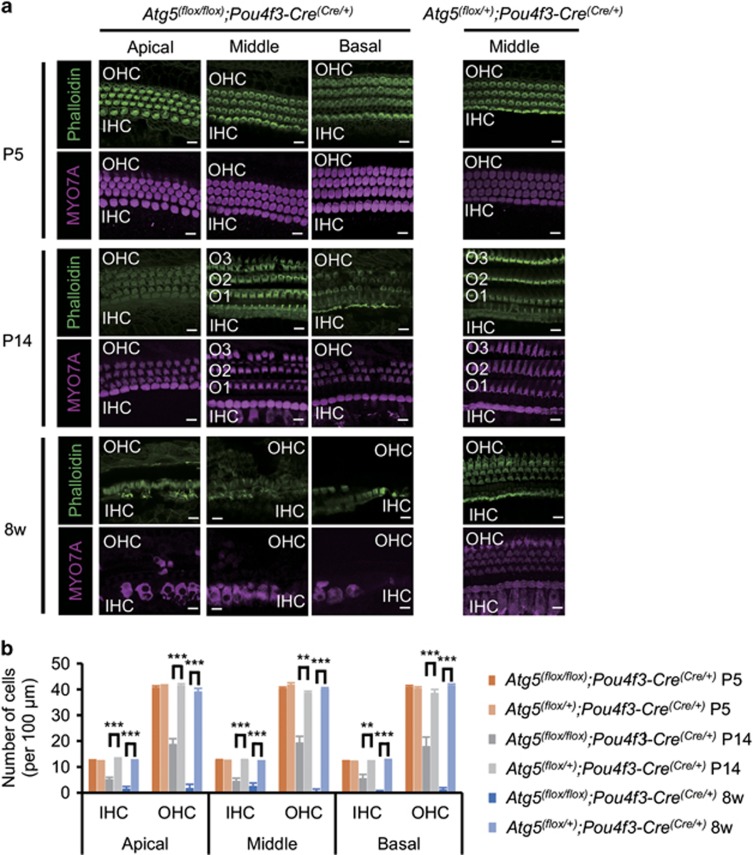
Damage to *Atg5*-deficient HCs was progressively developed. (**a**) Confocal imaging of the organ of Corti in *Atg5*^*flox/flox*^*;Pou4f3-Cre* and *Atg5*^*flox/+*^*;Pou4f3-Cre* mice. The HCs in *Atg5*^*flox/flox*^*;Pou4f3-Cre* mice showed normal morphogenesis at P5. At P14, stereocilia were damaged in many HCs and some of the outer HC (OHC) bodies were destroyed. At 8 weeks of age, almost all of the OHC bodies and many inner HC (IHC) bodies were destroyed, and most of the stereocilia in the IHCs were damaged. MYO7A and phalloidin were used as markers of the HC cytoplasm and stereocilia, respectively. O1, the first row outer HC; O2, the second row outer HC; O3, the third row outer HC. Scale bars: 10 *μ*m. (**b**) A comparison of the number of cells per 100 *μ*m from *Atg5*^*flox/flox*^*;Pou4f3-Cre* mice (*n*=5) and *Atg5*^*flox/+*^*;Pou4f3-Cre* mice (*n*=5) was performed by Student’s *t*-test at P5, P14, and 8 weeks of age. At P5, there were no significant differences between these genotypes. At P14 and 8 weeks of age, *Atg5*^*flox/flox*^*;Pou4f3-Cre* mice had significantly fewer HCs than *Atg5*^*flox/+*^*;Pou4f3-Cre* mice. Error bars: S.E. **P<0.01, ***P<0.001

**Figure 6:**
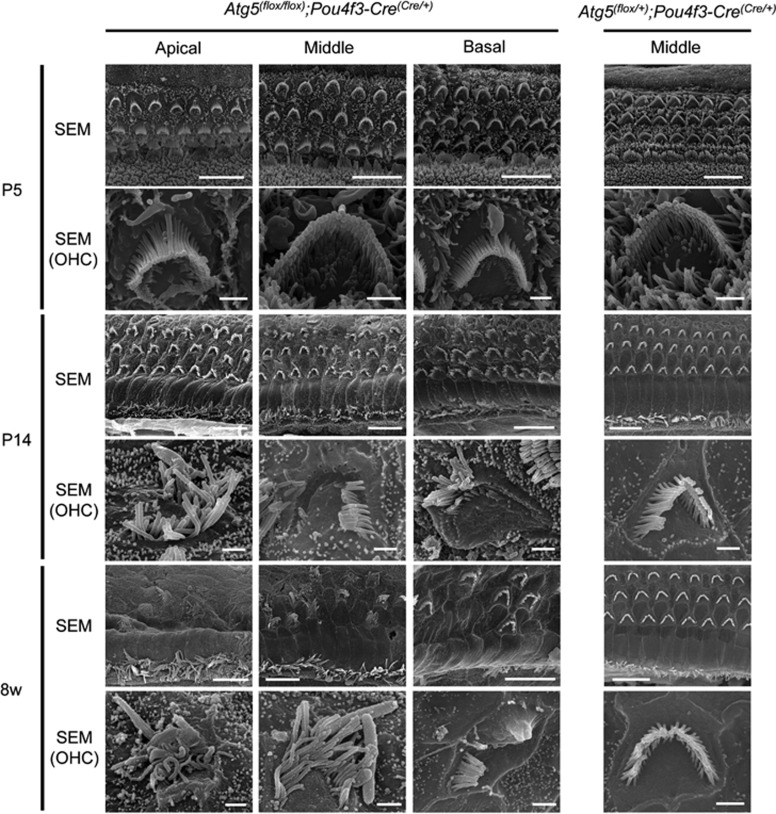
Stereocilia of *Atg5*-deficient HCs were progressively damaged. Scanning electron microscopy of the HCs in *Atg5*^*flox/flox*^*;Pou4f3-Cre* and *Atg5*^*flox/+*^*;Pou4f3-Cre* mice. The HCs in *Atg5*^*flox/flox*^*;Pou4f3-Cre* mice showed normal morphogenesis at P5. At P14, stereocilia were damaged or irregularly shaped in many HCs. At 8 weeks of age in *Atg5*^*flox/flox*^*;Pou4f3-Cre* mice, almost all of the stereocilia in the outer HCs (OHCs) were destroyed and most of the stereocilia in the inner HCs were damaged or irregularly shaped. Scale bars: 10 *μ*m for total HCs, 1 *μ*m for OHC

We then investigated the effect of *Atg5* on the morphogenesis of the auditory HCs. The HCs in *Atg5*^*flox/flox*^*;Pou4f3-Cre* mice had normal morphogenesis and no reduction in the number of HCs at P5 (Figures 5a and b). At P14, however, many stereocilia labeled with fluorescent phalloidin were damaged in both inner and outer HCs, and the body of the outer HCs was destroyed to some extent (Figure 5a). At 8 weeks of age, almost all the outer HC bodies were destroyed and most stereocilia and many cell bodies in the inner HCs were damaged. These findings suggest that the damage to stereocilia and cell bodies of the HCs in *Atg5*^*flox/flox*^*;Pou4f3-Cre* mice was progressively developed with age (Figure 5a). The number of normal HCs per 100 *μ*m of *Atg5*^*flox/flox*^*;Pou4f3-Cre* mice was significantly smaller compared with *Atg5*^*flox/+*^*;Pou4f3-Cre* mice at both P14 and 8 weeks of age (Figure 5b). Scanning electron microscopy showed that the stereocilia of *Atg5*^*flox/flox*^*;Pou4f3-Cre* mice looked normal at P5 but damaged or irregularly shaped at P14 (Figure 6 and Supplementary Figure S2). This damage progressed up to 8 weeks of age.

**Figure 7:**
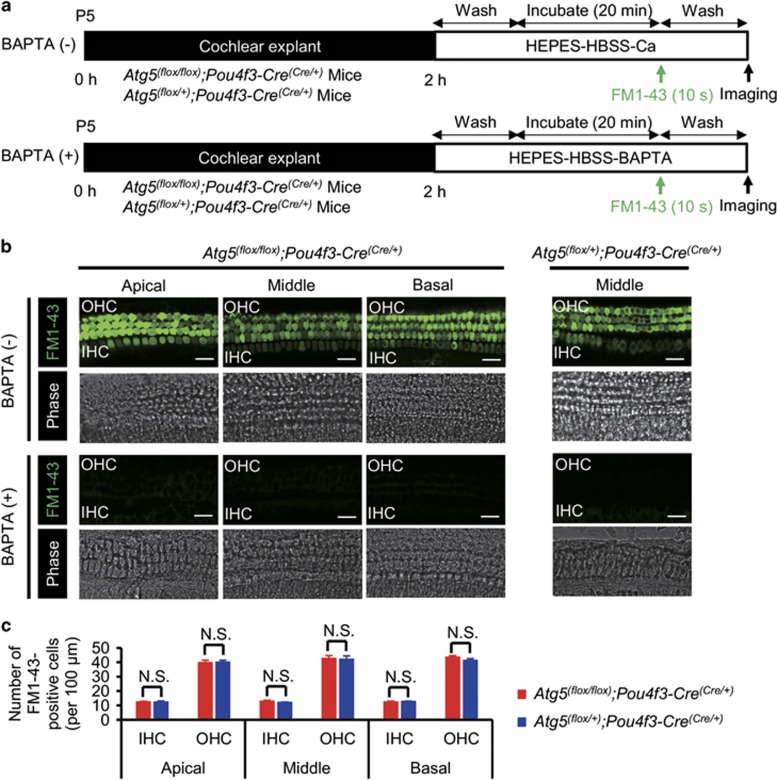
FM1-43 uptake into the HCs was not affected in P5 *Atg5*^*flox/flox*^*;Pou4f3-Cre* mice. (**a**) Experimental design of FM1-43 uptake assay in cochlear explant cultures established from P5 mice. FM1-43 uptake assay was performed after 2 h of incubation. (**b**) FM1-43 uptake in P5 cochlear explant cultures derived from *Atg5*^*flox/flox*^*;Pou4f3-Cre* and *Atg5*^*flox/+*^*;Pou4f3-Cre* mice. Both *Atg5*^*flox/flox*^*;Pou4f3-Cre* and *Atg5*^*flox/+*^*;Pou4f3-Cre* mice HCs displayed robust uptake of FM1-43, whereas both *Atg5*^*flox/flox*^*;Pou4f3-Cre* and *Atg5*^*flox/+*^*;Pou4f3-Cre* mice HCs pretreated with 5 mM BAPTA did not take up FM1-43. (**c**) A comparison of the number of FM1-43-postitive HCs per 100 *μ*m between *Atg5*^*flox/flox*^*;Pou4f3-Cre* mice (*n*=5) and *Atg5*^*flox/+*^*;Pou4f3-Cre* mice (*n*=5) was performed by Student’s *t*-test. There were no significant differences in the number of FM1-43-postitive HCs between *Atg5*^*flox/flox*^*;Pou4f3-Cre* and *Atg5*^*flox/+*^*;Pou4f3-Cre* mice. IHC, inner HC; OHC, outer HC. Scale bars: 20 *μ*m. Error bars: S.E. NS, not significant

**Figure 8:**
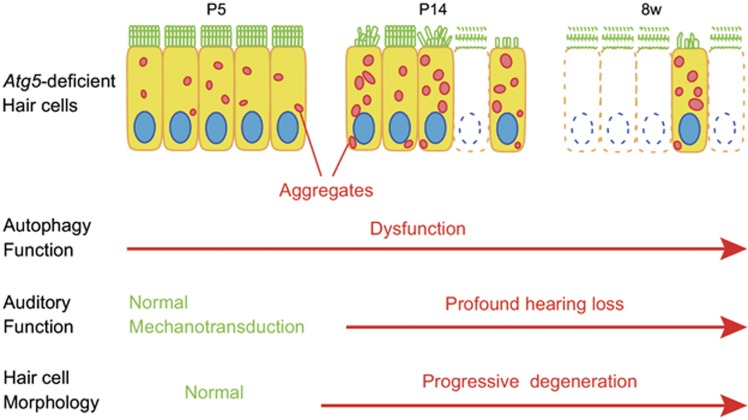
Schematic view of the phenotype in the HCs of *Atg5*^*flox/flox*^*;Pou4f3-Cre* mice based on morphological and functional analyses. In P5 HCs, no morphological changes were observed and mechanotransduction was normal, although aggregates containing ubiquitin and p62 were observed in the cytoplasm. At P14, aggregates became more massive. Stereocilia were damaged in many HCs and some of HC bodies were destroyed. P14 mice showed profound hearing loss. At 8 weeks of age, loss of HCs was progressively developed

The process of mechanotransduction in the auditory HCs is initiated by the opening of cation channels located at the tips of the stereocilia, which can be assessed visually by uptake of the fluorescent styryl dye FM1-43.^16, 17^ To evaluate the function of morphologically normal neonatal HCs in *Atg5*^*flox/flox*^*;Pou4f3-Cre* mice before morphological degeneration, we performed an FM1-43 uptake assay using P5 cochlear explant cultures (Figure 7a). FM1-43 uptake into the HCs was not affected in P5 *Atg5*^*flox/flox*^*;Pou4f3-Cre* mice (Figures 7b and c), indicating that the HCs of the *Atg5*^*flox/flox*^*;Pou4f3-Cre* mice develop normally and have functioning mechanotransduction channels at least until P5. These results suggest that the cause of hearing loss in *Atg5*^*flox/flox*^*;Pou4f3-Cre* mice is degeneration, rather than maldevelopment, of auditory HCs and that *Atg5* is required for maintenance of morphology of these cells (Figure 8).

## Discussion

We revealed that mice deficient in *Atg5* in auditory HCs showed profound congenital hearing loss and progressive degeneration of these cells. Polyubiquitinated proteins and p62 accumulated in autophagy-deficient HCs as inclusion bodies during the first postnatal week and these aggregates increased in number. Auditory HCs are postmitotic and do not regenerate after their loss in mammals. The effect of the loss of basal autophagy, which is important for homeostasis of postmitotic cells, on hearing acuity has been unknown, although autophagy-related 4b (*Atg4b*), a gene essential for autophagy, is necessary for the development of otoconia in the murine vestibular system.^18^ Our study first revealed the contribution of basal autophagy to intracellular quality control in HCs and hearing acuity in mammals.

Recently, several studies showed that autophagy is involved in the prevention of auditory damage.^19, 20, 21^ According to an *in vitro* study using a murine auditory cell line, the House Ear Institute-Organ of Corti 1 cell line, suppression of autophagy by *Atg7* (autophagy-related 7) knockdown decreased cell viability in H_2_O_2_-induced cell death, whereas activation of autophagy by rapamycin protected against H_2_O_2_-induced cell death.^20^ A previous study in rats revealed that treatment with rapamycin, which is an autophagy activator, significantly attenuated cisplatin-induced hearing loss, decreased the level of malondialdehyde (an oxidative stress marker), and alleviated HC damage.^19^ Another study revealed that treatment of mice with rapamycin significantly increased LC3B expression, decreased the levels of the oxidative stress markers 4-hydroxynonenal (4-HNE) and 3-nitrotyrosine (3-NT), reduced noise-induced cell loss in the outer HCs, and reduced the amount of hearing loss.^21^ Conversely, treatment with 3-methyladenine, an autophagy inhibitor, or LC3B siRNA reduced LC3B expression, increased 4-HNE and 3-NT levels, and exacerbated temporary-to-permanent threshold shift.

Hearing loss has recently been reported in an autophagy-related disease, Vici syndrome.^22, 23, 24^ This syndrome, which is due to recessive mutations in the *EPG5* gene encoding ectopic P granules protein 5 (EPG5), a key autophagy regulator, is a congenital multisystem disorder characterized by corpus callosal agenesis, cataracts, cardiomyopathy, oculocutaneous hypopigmentation, and immunodeficiency. Life expectancy of patients in Vici syndrome is severely reduced. Sensorineural hearing loss has subsequently been reported in several cases of Vici syndrome with or without confirmed EPG5 mutations,^22, 23, 24^ although the detailed mechanisms underlying the sensorineural hearing loss have not been elucidated.

In mice, cochlear HC stereocilia continue to develop in a well-organized process during the early postnatal weeks.^25, 26^ The auditory ribbon synapses also form and mature after birth.^27, 28^ The external auditory canal remains closed until P12, and mice do not respond to air-conducted sound until then.^29^ Therefore, it has been considered that the onset of hearing occurs around P12. In fact, ABRs cannot be reliably recorded before P12–P14.^29, 30, 31^ In the present study, the morphology and mechanotransduction of *Atg5*-deficient auditory HCs were both normal in P5, although polyubiquitinated proteins and p62 were already accumulated. At P14, however, mice deficient in *Atg5* in auditory HCs displayed progressive accumulation of polyubiquitinated protein aggregates in the HCs, as well as HC degeneration and profound hearing loss. These results strongly suggest the importance of basal autophagy maintaining the morphology of auditory HCs during the early postnatal stage until the maturation of the auditory system.

*Pou4f3* is also expressed in the retinal ganglion cells (RGCs), and *Atg5* might be deficient in RGCs in *Atg5*^*flox/flox*^*;Pou4f3-Cre* mice. We have not analyzed the role of *Atg5* on RGCs using these mice. However, a previously study examined the effects of downregulation of *Atg5* expression in RGCs by RGC-specific transduction using adeno-associated virus serotype 2 (AAV2)^32^ and demonstrated that the downregulation of *Atg5* in RGCs made RGCs more vulnerable to optic nerve axotomy. Fewer surviving RGCs were observed postaxotomy in *Atg5*^*flox/flox*^ mice injected with AAV2-GFP-Cre in comparison with those injected with AAV2-GFP control vector.

The relationship between p62 accumulation and pathogenesis of disease has previously been well discussed.^15, 33, 34, 35^ p62 has been identified as a common component of ubiquitin-containing protein aggregates, known as inclusion bodies in alcoholic hepatitis and steatohepatitis.^35^ The inclusion bodies have also been found in neurodegenerative diseases, such as Parkinson’s disease and amyotrophic lateral sclerosis.^33, 34^ Reduction of autophagic activity might contribute to the generation of inclusion bodies in these diseases. The hepatic damage in *Atg7* knockout mice was ameliorated by simultaneous genetic deletion of p62.^15^ However, the effect of the simultaneous deletion of p62 on neurodegeneration was very small, indicating that the contribution of p62 accumulation associated with autophagy suppression on the pathogenesis of disease is cell-type specific.^15^ In the present study, both cell damage and auditory dysfunction in mice deficient in *Atg5* in auditory HCs increased with the exacerbation of p62 accumulation. p62 accumulation, therefore, might have an important role in the progression of damage in autophagy-deficient HCs.

## Materials and methods

### Animals

Experimental procedures to produce *Atg5*^*flox/flox*^,^12^ *Pou4f3-Cre* transgenic,^13^ and GFP-LC3 transgenic mice^11^ have been described previously. Genotyping for *Atg5*^*flox/flox*^,^12^ Cre,^13^ and GFP-LC3^36^ was conducted as described. Wild-type C57BL/6 mice were obtained from Japan CLEA (Tokyo, Japan). All mice were given free access to food. All animal experiments were performed in accordance with the institutional guidelines of the Animal Care and Use Committees of the University of Tokyo (no. P11-100, P12-77, P13-086) and the National Institutes of Health Guidelines for the Care and Use of Laboratory Animals.

### Antibodies and fluorescent probes

We purchased the following primary antibodies: chicken polyclonal antibodies against GFP (Abcam, Cambridge, MA, USA), rabbit polyclonal antibodies against MYO7A (Proteus Biosciences, Ramona, CA, USA), mouse monoclonal antibodies against ubiquitin 1B3 (MBL, Nagoya, Japan), and guinea pig polyclonal antibody against p62 (PROGEN, Heidelberg, Germany). We also purchased Alexa 488-phalloidin and the following secondary antibodies from Molecular Probes (Eugene, OR, USA): Alexa 488-conjugated goat anti-chicken IgG, Alexa 488-conjugated goat anti-mouse IgG, Alexa 568-conjugated goat anti-rabbit IgG, Alexa 568-conjugated goat anti-guinea pig IgG, and Alexa 680-conjugated goat anti-rabbit IgG.

### Immunohistochemical analyses of cochlear explant cultures

P5 GFP-LC3 transgenic mice were anesthetized and decapitated. The temporal bones were dissected, and the cochleae were freed from the surrounding tissue. After removing the lateral wall of the cochlea, the organ of Corti was dissected from the cochlear modiolus. Tissue samples were then placed on glass-mesh inserts (Falcon, Franklin Lakes, NJ, USA) and cultured in serum-free modified Eagle’s medium (MEM; Invitrogen, Waltham, MA, USA), supplemented with 3 g/l glucose (Wako Pure Chemicals, Osaka, Japan) and 0.3 g/l penicillin G (Wako Pure Chemicals), at 37 °C for 4 h in a humidified incubator with 95% air and 5% atmospheric carbon dioxide. For tissue samples with lysosomal protease inhibitors, E64d (10 *μ*g/ml) and pepstatin A (10 *μ*g/ml) were added to the above-mentioned medium. After the culture was finished, the specimens were fixed in 4% paraformaldehyde (PFA) in phosphate-buffered saline (PBS; pH 7.4). For immunohistochemistry, after blocking treatment with IMAGE-IT FX (Thermo Fisher Scientific, Waltham, MA, USA) at 37 °C for 30 min, the samples were incubated with primary antibodies at 4 °C overnight. The samples were then incubated with secondary antibodies at 37 °C for 30 min and mounted for analysis using an A1R confocal laser scanning microscope (Nikon, Tokyo, Japan). The GFP-LC3 puncta formation assay was performed in the cytoplasm of the HCs with the focal plane at the apical body of the middle cochlear turn. The average number of punctate structures per cell was calculated from 5 inner and 15 outer HCs per experiment. Quantification of GFP-LC3-associated autophagosomes was performed with ImageJ (NIH, Bethesda, MD, USA), using the basal dispersed GFP-LC3 fluorescence as the threshold.

### Cochlear surface preparation for immunohistochemistry

The cochleae were fixed in 4% PFA in PBS. The surface of the organ of Corti was prepared by removing the bony otic capsule, the stria vascularis, the Reissner's membrane, and the tectorial membrane. For immunohistochemistry, the specimens were incubated with primary antibodies at 4 °C overnight. The specimens were then incubated with secondary antibodies at 37 °C for 30 min and mounted for analysis with the A1R confocal laser scanning microscope.

### Histological and immunohistochemical analyses for paraffin-embedded sections

The cochleae were fixed in 4% PFA in PBS and decalcified in 10% ethylenediamine tetraacetic acid for several days. Then the specimens were dehydrated in a graded ethanol series and embedded in paraffin. The paraffin block containing the tissue was cut into 4-*μ*m sections, and the sections were deparaffinized and then rehydrated through a xylene and ethanol series. For immunohistochemistry, after antigen retrieval and blocking treatment with 0.1% Triton X-100 and 5% goat serum in PBS at room temperature for 30 min, sections were incubated with primary antibodies at 4 °C overnight. The sections were then incubated with secondary antibodies at 37 °C for 30 min and mounted for analysis with the A1R confocal laser scanning microscope. In all images, the same threshold fluorescence intensity for each channel was fixed. An inner HC and three outer HCs were selected from each cochlear turn (i.e., the apical turn, the middle turn, or the basal turn), and the number of aggregates containing ubiquitin and p62 per 100-*μ*m^2^ field of the cytoplasm in the auditory HCs, the outer HCs, and the total HCs was then counted for each cochlea. For each mouse group (i.e., P5 *Atg5*^*flox/flox*^*;Pou4f3-Cre* mice, P5 *Atg5*^*flox/+*^*;Pou4f3-Cre* mice, P14 *Atg5*^*flox/flox*^*;Pou4f3-Cre* mice, or P14 *Atg5*^*flox/+*^*;Pou4f3-Cre* mice), six cochleae were used for a comparison of the number of aggregates.

### Scanning electron microscopy

The temporal bones were fixed with 2.5% glutaraldehyde and 2% PFA in 0.1 M phosphate buffer (pH 7.4) at room temperature for 1 h. The surface of the organ of Corti was prepared by removing the bony otic capsule, the stria vascularis, the Reissner's membrane, and the tectorial membrane. The tissues were postfixed with 1% osmium tetroxide buffered with 0.1 M phosphate buffer, dehydrated in a graded ethanol series, critical-point dried, and sputter-coated with platinum. The samples were observed under a scanning electron microscope (S-4500, Hitachi, Tokyo, Japan).

### Auditory brainstem response

ABR was evaluated at P14, 4 weeks of age, and 8 weeks of age as previously described.^37^ Before the ABR measurements, the external auditory canals and tympanic membranes were confirmed to be normal in all mice. Subdermal electrodes were placed on the vertex (active electrode), in the postauricular area of the measured ear (reference electrode), and in the postauricular area of the opposite ear (ground electrode). The speaker was placed 10 cm from the tragus of the stimulated ear. A tone-burst sound (2, 4, 8, 16, and 32 kHz) was produced by a sound stimulator (Neuropack MEB-2200, Nihon Kohden, Tokyo, Japan). The stimulus duration was 15 ms, the presentation rate was 11/s, and the rise/fall time was 1 ms. At each stimulus level, ABR was obtaind by averaging 500 responses. The threshold of ABR was determined as the lowest intensity level at which a clear reproducible waveform was obtained in the trace. If no response was obtained, we used 100 dB sound pressure level as the threshold value volume for further calculations.

### FM1-43 uptake

Cochleae were dissected from P5 mice in PBS and explanted onto *μ*-Slide eight-well tissue culture plates (Ibidi, Martinsried, Germany). Explants were incubated in MEM (Invitrogen), supplemented with 3 g/l glucose (Wako Pure Chemicals) and 0.3 g/l penicillin G (Wako Pure Chemicals), at 37 °C for 2 h in a humidified incubator with 95% air and 5% atmospheric carbon dioxide. FM1-43 (Life Technologies, Waltham, MA, USA) was applied to the mounted tissue according to a previously described method.^38^ Explants were washed three times with Hanks balanced salt solution (HBSS) (Nacalai Tesque, Kyoto, Japan) containing 10 mM 4-(2-hydroxyethyl)-1-piperazineethanesulfonic acid (HEPES) buffer and 1.3 mM Ca^2+^ (HEPES-HBSS-Ca). After an additional 20-min incubation in HEPES-HBSS-Ca, 5 *μ*M FM1-43 in HEPES-HBSS was applied for 10 s at room temperature, followed immediately by 4 washes (within 1 min) with HEPES-HBSS-Ca. When testing the effects of 1,2-bis-(2-aminophenoxyethane)-N,N,N′,N′-tetraacetic acid (BAPTA), HEPES-HBSS-Ca was replaced with HEPES-HBSS containing 5 mM BAPTA at every step. FM1-43 fluorescence of magnified images from the cochleae was captured with a 40 × objective lens 5 min after FM1-43 treatment. Images were obtained with the A1R confocal laser scanning microscope.

### Statistical analyses

Data are expressed as the mean±S.E. Statistical analyses were conducted by using IBM SPSS statistics version 22 (IBM, Tokyo, Japan) or R version 3.0.2 (R Development Core Team, Auckland, New Zealand). A comparison of the number of punctate structures between *Atg5*^*flox/flox*^*;Pou4f3-Cre* and *Atg5*^*flox/+*^*;Pou4f3-Cre* mice was performed by Student’s *t*-test. For a comparison of the number of aggregates containing ubiquitin and p62, two-way analysis of variance was performed with genotype × age as variables, followed by Bonferroni’s *post hoc* tests, wherever appropriate. A comparison of the ABR threshold between *Atg5*^*flox/flox*^*;Pou4f3-Cre* and *Atg5*^*flox/+*^*;Pou4f3-Cre* mice was performed by Student’s *t*-test. A comparison of the number of cells per 100 *μ*m between *Atg5*^*flox/flox*^*;Pou4f3-Cre* mice (*n*=5) and *Atg5*^*flox/+*^*;Pou4f3-Cre* mice (*n*=5) was performed by Student’s *t*-test. A comparison of the number of FM1-43-postitive HCs per 100 *μ*m between *Atg5*^*flox/flox*^*;Pou4f3-Cre* and *Atg5*^*flox/+*^*;Pou4f3-Cre* mice was performed by Student’s *t*-test. *P*<0.05 was considered statistically significant.

## References

[bib1] Mathers C, Smith A, Concha MGlobal Burden of Hearing Loss in the Year 2000. Global Burden of Disease: Geneva, Switzerland, pp 1–30, 2003.

[bib2] World Health Organizaton. Deafness and hearing loss. Available at http://www.who.int/mediacentre/factsheets/fs300/en.

[bib3] Edge AS, Chen ZY. Hair cell regeneration. Curr Opin Neurobiol 2008; 18: 377–382. 18929656 10.1016/j.conb.2008.10.001 PMC5653255

[bib4] Kelley MW. Regulation of cell fate in the sensory epithelia of the inner ear. Nat Rev Neurosci 2006; 7: 837–849. 17053809 10.1038/nrn1987

[bib5] Fujioka M, Okano H, Edge AS. Manipulating cell fate in the cochlea: a feasible therapy for hearing loss. Trends Neurosci 2015; 38: 139–144. 25593106 10.1016/j.tins.2014.12.004 PMC4352405

[bib6] Izumikawa M, Minoda R, Kawamoto K, Abrashkin KA, Swiderski DL, Dolan DF et al. Auditory hair cell replacement and hearing improvement by Atoh1 gene therapy in deaf mammals. Nat Med 2005; 11: 271–276. 15711559 10.1038/nm1193

[bib7] Mizutari K, Fujioka M, Hosoya M, Bramhall N, Okano HJ, Okano H et al. Notch inhibition induces cochlear hair cell regeneration and recovery of hearing after acoustic trauma. Neuron 2013; 77: 58–69. 23312516 10.1016/j.neuron.2012.10.032 PMC3573859

[bib8] Mizushima N, Komatsu M. Autophagy: renovation of cells and tissues. Cell 2011; 147: 728–741. 22078875 10.1016/j.cell.2011.10.026

[bib9] Levine B, Kroemer G. Autophagy in the pathogenesis of disease. Cell 2008; 132: 27–42. 18191218 10.1016/j.cell.2007.12.018 PMC2696814

[bib10] Mizushima N, Yoshimori T, Ohsumi Y. The role of Atg proteins in autophagosome formation. Annu Rev Cell Dev Biol 2011; 27: 107–132. 21801009 10.1146/annurev-cellbio-092910-154005

[bib11] Mizushima N, Yamamoto A, Matsui M, Yoshimori T, Ohsumi Y. *In vivo* analysis of autophagy in response to nutrient starvation using transgenic mice expressing a fluorescent autophagosome marker. Mol Biol Cell 2004; 15: 1101–1111. 14699058 10.1091/mbc.E03-09-0704 PMC363084

[bib12] Hara T, Nakamura K, Matsui M, Yamamoto A, Nakahara Y, Suzuki-Migishima R et al. Suppression of basal autophagy in neural cells causes neurodegenerative disease in mice. Nature 2006; 441: 885–889. 16625204 10.1038/nature04724

[bib13] Sage C, Huang M, Vollrath MA, Brown MC, Hinds PW, Corey DP et al. Essential role of retinoblastoma protein in mammalian hair cell development and hearing. Proc Natl Acad Sci USA 2006; 103: 7345–7350. 16648263 10.1073/pnas.0510631103 PMC1450112

[bib14] Bjorkoy G, Lamark T, Brech A, Outzen H, Perander M, Overvatn A et al. p62/SQSTM1 forms protein aggregates degraded by autophagy and has a protective effect on huntingtin-induced cell death. J Cell Biol 2005; 171: 603–614. 16286508 10.1083/jcb.200507002 PMC2171557

[bib15] Komatsu M, Waguri S, Koike M, Sou YS, Ueno T, Hara T et al. Homeostatic levels of p62 control cytoplasmic inclusion body formation in autophagy-deficient mice. Cell 2007; 131: 1149–1163. 18083104 10.1016/j.cell.2007.10.035

[bib16] Gale JE, Marcotti W, Kennedy HJ, Kros CJ, Richardson GP. FM1-43 dye behaves as a permeant blocker of the hair-cell mechanotransducer channel. J Neurosci 2001; 21: 7013–7025. 11549711 10.1523/JNEUROSCI.21-18-07013.2001 PMC6762973

[bib17] Meyers JR, MacDonald RB, Duggan A, Lenzi D, Standaert DG, Corwin JT et al. Lighting up the senses: FM1-43 loading of sensory cells through nonselective ion channels. J Neurosci 2003; 23: 4054–4065. 12764092 10.1523/JNEUROSCI.23-10-04054.2003 PMC6741082

[bib18] Marino G, Fernandez AF, Cabrera S, Lundberg YW, Cabanillas R, Rodriguez F et al. Autophagy is essential for mouse sense of balance. J Clin Invest 2010; 120: 2331–2344. 20577052 10.1172/JCI42601 PMC2898610

[bib19] Fang B, Xiao H. Rapamycin alleviates cisplatin-induced ototoxicity *in vivo*. Biochem Biophys Res Commun 2014; 448: 443–447. 24796670 10.1016/j.bbrc.2014.04.123

[bib20] Hayashi K, Dan K, Goto F, Tshuchihashi N, Nomura Y, Fujioka M et al. The autophagy pathway maintained signaling crosstalk with the Keap1-Nrf2 system through p62 in auditory cells under oxidative stress. Cell Signal 2015; 27: 382–393. 25435427 10.1016/j.cellsig.2014.11.024

[bib21] Yuan H, Wang X, Hill K, Chen J, Lemasters J, Yang SM et al. Autophagy attenuates noise-induced hearing loss by reducing oxidative stress. Antioxid Redox Signal 2015; 22: 1308–1324. 25694169 10.1089/ars.2014.6004 PMC4410759

[bib22] Finocchi A, Angelino G, Cantarutti N, Corbari M, Bevivino E, Cascioli S et al. Immunodeficiency in Vici syndrome: a heterogeneous phenotype. Am J Med Genet A 2012; 158A: 434–439. 21965116 10.1002/ajmg.a.34244

[bib23] McClelland V, Cullup T, Bodi I, Ruddy D, Buj-Bello A, Biancalana V et al. Vici syndrome associated with sensorineural hearing loss and evidence of neuromuscular involvement on muscle biopsy. Am J Med Genet A 2010; 152A: 741–747. 20186778 10.1002/ajmg.a.33296

[bib24] Ozkale M, Erol I, Gumus A, Ozkale Y, Alehan F. Vici syndrome associated with sensorineural hearing loss and laryngomalacia. Pediatr Neurol 2012; 47: 375–378. 23044023 10.1016/j.pediatrneurol.2012.07.007

[bib25] Jones C, Chen P. Primary cilia in planar cell polarity regulation of the inner ear. Curr Top Dev Biol 2008; 85: 197–224. 19147007 10.1016/S0070-2153(08)00808-9 PMC4158840

[bib26] Petit C, Richardson GP. Linking genes underlying deafness to hair-bundle development and function. Nat Neurosci 2009; 12: 703–710. 19471269 10.1038/nn.2330 PMC3332156

[bib27] Maison SF, Adams JC, Liberman MC. Olivocochlear innervation in the mouse: immunocytochemical maps, crossed versus uncrossed contributions, and transmitter colocalization. J Comp Neurol 2003; 455: 406–416. 12483691 10.1002/cne.10490 PMC1805785

[bib28] Sobkowicz HM, Rose JE, Scott GE, Slapnick SM. Ribbon synapses in the developing intact and cultured organ of Corti in the mouse. J Neurosci 1982; 2: 942–957. 7097321 10.1523/JNEUROSCI.02-07-00942.1982 PMC6564385

[bib29] Uziel A, Romand R, Marot M. Development of cochlear potentials in rats. Audiology 1981; 20: 89–100. 7224981 10.3109/00206098109072687

[bib30] Blatchley BJ, Cooper WA, Coleman JR. Development of auditory brainstem response to tone pip stimuli in the rat. Brain Res 1987; 429: 75–84. 3567661 10.1016/0165-3806(87)90140-4

[bib31] Geal-Dor M, Freeman S, Li G, Sohmer H. Development of hearing in neonatal rats: air and bone conducted ABR thresholds. Hear Res 1993; 69: 236–242. 8226345 10.1016/0378-5955(93)90113-f

[bib32] Rodriguez-Muela N, Germain F, Marino G, Fitze PS, Boya P. Autophagy promotes survival of retinal ganglion cells after optic nerve axotomy in mice. Cell Death Differ 2012; 19: 162–169. 21701497 10.1038/cdd.2011.88 PMC3252838

[bib33] Kuusisto E, Salminen A, Alafuzoff I. Ubiquitin-binding protein p62 is present in neuronal and glial inclusions in human tauopathies and synucleinopathies. Neuroreport 2001; 12: 2085–2090. 11447312 10.1097/00001756-200107200-00009

[bib34] Nakano T, Nakaso K, Nakashima K, Ohama E. Expression of ubiquitin-binding protein p62 in ubiquitin-immunoreactive intraneuronal inclusions in amyotrophic lateral sclerosis with dementia: analysis of five autopsy cases with broad clinicopathological spectrum. Acta Neuropathol 2004; 107: 359–364. 14762676 10.1007/s00401-004-0821-7

[bib35] Stumptner C, Fuchsbichler A, Heid H, Zatloukal K, Denk H. Mallory body—a disease-associated type of sequestosome. Hepatology 2002; 35: 1053–1062. 11981755 10.1053/jhep.2002.32674

[bib36] Kuma A, Mizushima N. Chromosomal mapping of the GFP-LC3 transgene in GFP-LC3 mice. Autophagy 2008; 4: 61–62. 17786029 10.4161/auto.4846

[bib37] Fujimoto C, Ozeki H, Uchijima Y, Suzukawa K, Mitani A, Fukuhara S et al. Establishment of mice expressing EGFP in the placode-derived inner ear sensory cell lineage and FACS-array analysis focused on the regional specificity of the otocyst. J Comp Neurol 2010; 518: 4702–4722. 20963824 10.1002/cne.22468

[bib38] Kawashima Y, Geleoc GS, Kurima K, Labay V, Lelli A, Asai Y et al. Mechanotransduction in mouse inner ear hair cells requires transmembrane channel-like genes. J Clin Invest 2011; 121: 4796–4809. 22105175 10.1172/JCI60405 PMC3223072

