# Supplementary figures and images for: Autophagy is essential for hearing in mice
Source: Cell Death Dis. 2017 May 11;8(5):e2780–. doi: 10.1038/cddis.2017.194 (PMC5520715; doi:10.1038/cddis.2017.194)

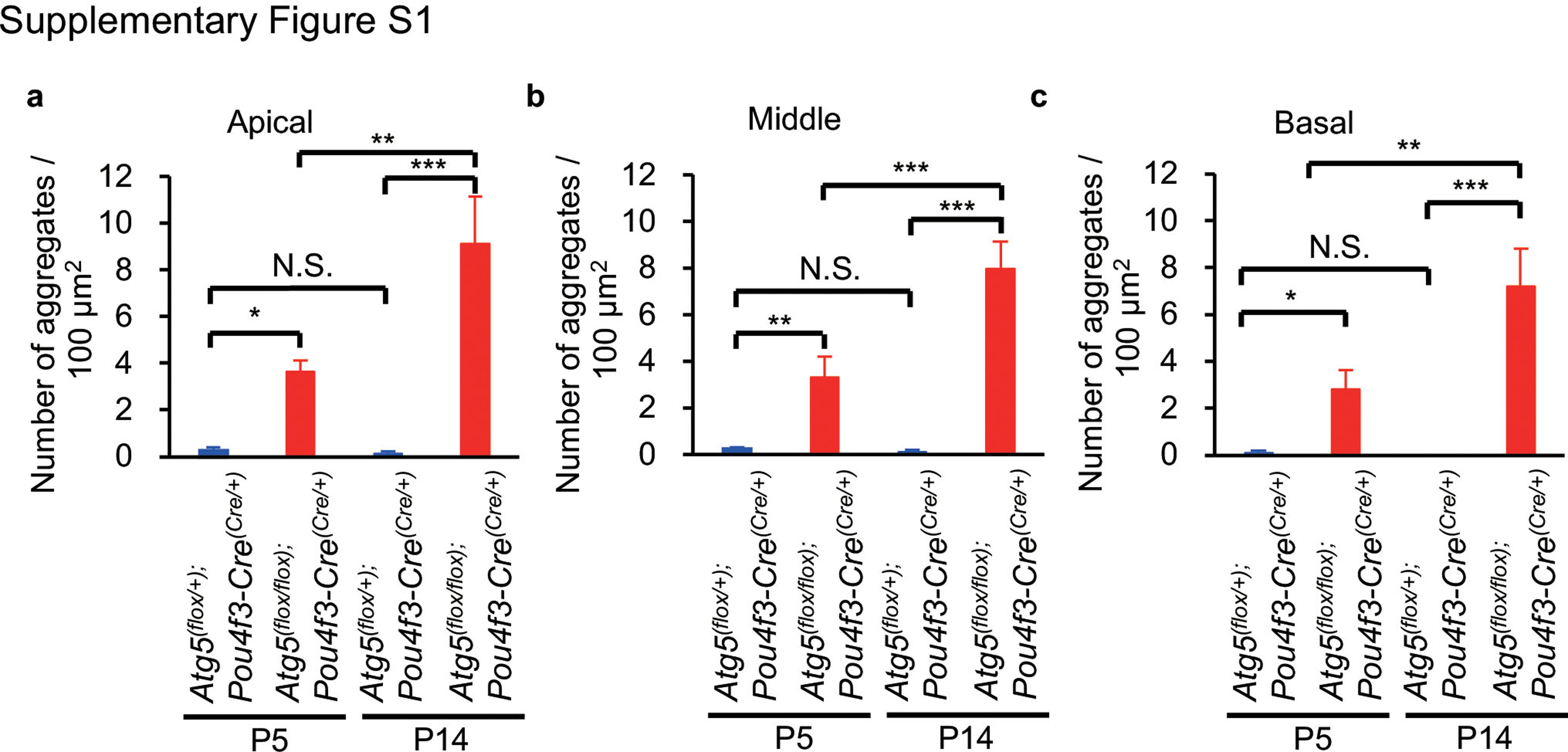

Supplement: Supplementary Figure S1 [file cddis2017194x1.tif]

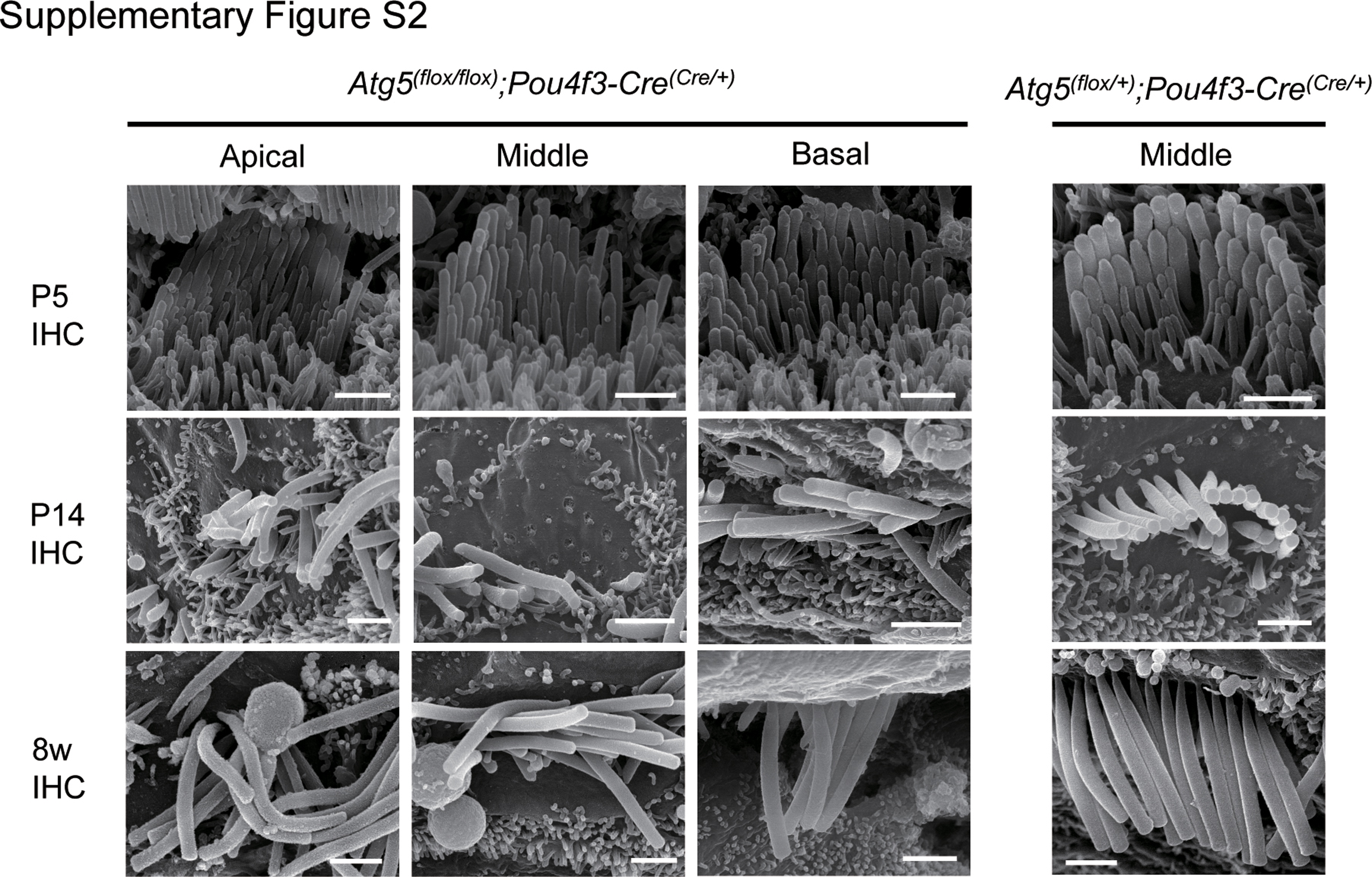

Supplement: Supplementary Figure S2 [file cddis2017194x2.tif]
